# Epigenetics and *In Utero* Acquired Predisposition to Metabolic Disease

**DOI:** 10.3389/fgene.2019.01270

**Published:** 2020-01-29

**Authors:** Annalisa Deodati, Elena Inzaghi, Stefano Cianfarani

**Affiliations:** ^1^Dipartimento Pediatrico Universitario Ospedaliero “Bambino Gesù” Children’s Hospital, Tor Vergata University, Rome, Italy; ^2^Department of Women’s and Children’s Health, Karolinska Institutet, Stockholm, Sweden

**Keywords:** intrauterine growth retardation, epigenetics, miRNAs, programming, cardiometabolic disease

## Abstract

Epidemiological evidence has shown an association between prenatal malnutrition and a higher risk of developing metabolic disease in adult life. An inadequate intrauterine milieu affects both growth and development, leading to a permanent programming of endocrine and metabolic functions. Programming may be due to the epigenetic modification of genes implicated in the regulation of key metabolic mechanisms, including DNA methylation, histone modifications, and microRNAs (miRNAs). The expression of miRNAs in organs that play a key role in metabolism is influenced by *in utero* programming, as demonstrated by both experimental and human studies. miRNAs modulate multiple pathways such as insulin signaling, immune responses, adipokine function, lipid metabolism, and food intake. Liver is one of the main target organs of programming, undergoing structural, functional, and epigenetic changes following the exposure to a suboptimal intrauterine environment. The focus of this review is to provide an overview of the effects of exposure to an adverse *in utero* milieu on epigenome with a focus on the molecular mechanisms involved in liver programming.

## Introduction

Early life events are associated with susceptibility to chronic diseases in adult life (Gluckman et al., 2005). Several studies have shown a clear link between the exposure to a suboptimal *in utero* environment leading to intrauterine growth retardation (IUGR) and the development of cardiometabolic disease in adulthood (Barker and Osmond, 1986; Barker et al., 1989). During the last three decades, mounting evidence has linked early exposure to malnutrition, epigenetic changes, and diseases, leading to the formulation of the “developmental origins of health and disease” (DOHaD) hypothesis (Barker, 2007). According to DOHaD the organism exposed to *in utero* undernourishment diverts the restricted nutrients to preserve growth and function of vital organs, such as brain, at the expense of growth and organs, such as liver and pancreas. This intrauterine adaptation is favorable for survival if the fetus is born in conditions of inadequate nutrition (Hales et al., 2001). However, detrimental consequences of developmental programming arise if the fetus is born in an environment with normal or even increased nutrient supply. The mismatch between pre- and postnatal environment may predispose the offspring to the development of cardiometabolic disease in adulthood (Gluckman et al., 2009).

Intrauterine programming occurs at “critical time windows” of fetal growth, characterized by a high rate of differentiation and/or proliferation, and involve genes, cells, tissues, and even whole organs (Fowden et al., 2006). Programming is an adaptive response of the organism to the surrounding environment: this capacity named “developmental plasticity” permits the development of a spectrum of phenotypes from a single genotype (Lucas et al., 1991; Gluckman and Hanson, 2004). When the resulting phenotype matches the environment, the organism will preserve a health status. On the contrary, if a mismatch occurs between the adaptive response and the environment, the organism is unable to cope with the environmental challenges, ultimately becoming susceptible to cardiometabolic disease (Godfrey et al., 2007).

One of the mechanisms of programming is the epigenetic change of genes involved in critical metabolic pathways. There is strong experimental evidence indicating that specific epigenetic hallmarks represent a sort of fingerprints of intrauterine programming. In this context, liver is a major target of *in utero* programming, undergoing structural, functional, and epigenetic changes as a result of early exposure to an adverse environment.

### “Programming”

**Figure 1:**
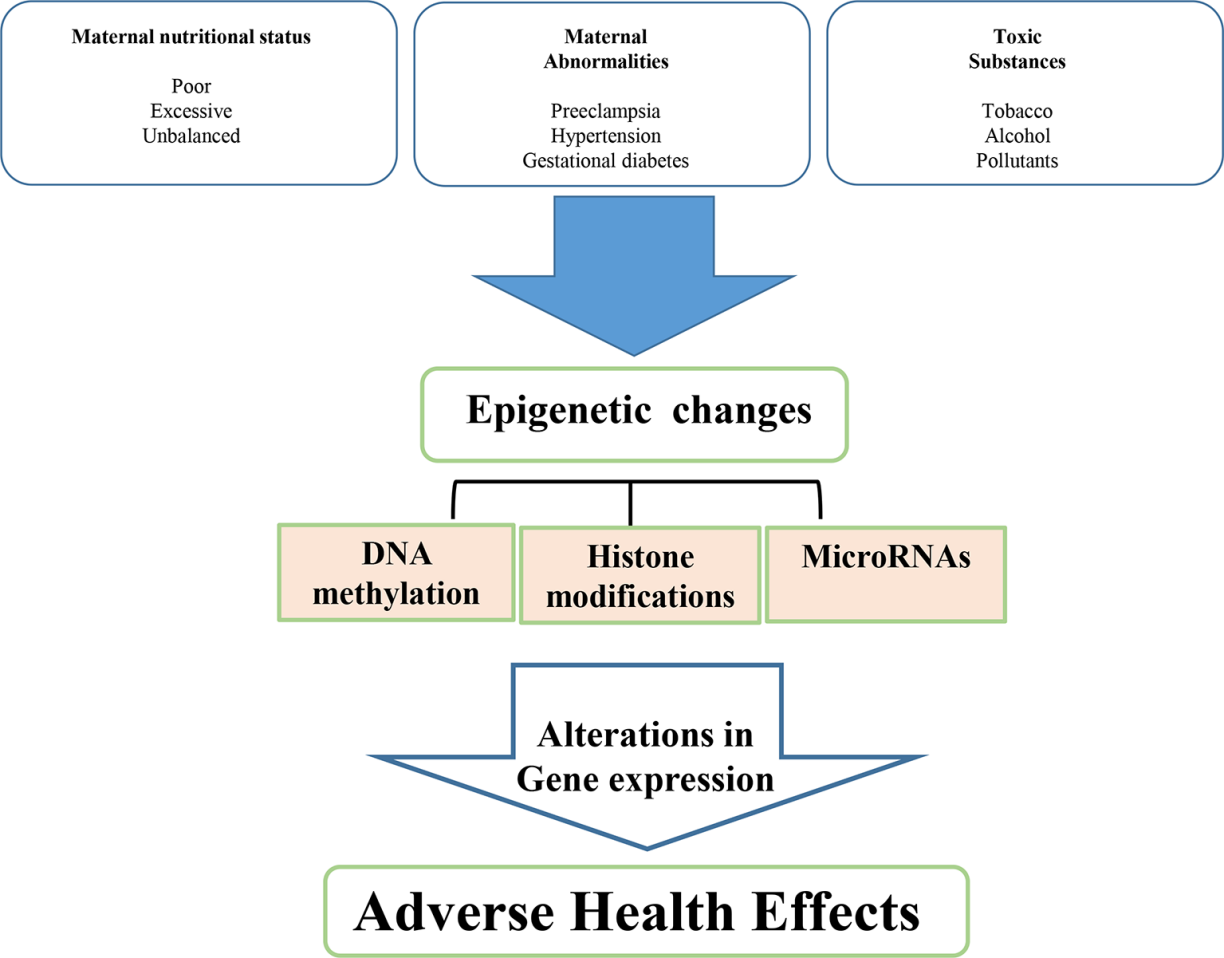
Environmental determinants related to intrauterine development resulting in low birth weight and metabolic disease in adult life.

Epigenetic regulation is a mechanism of programming and is basically related to gene silencing, genomic imprinting and transcriptional regulation of tissue-specific genes during cellular differentiation (Schubeler et al., 2000). The epigenetic control of gene expression depends on the modulation of chromatin structure and accessibility to transcription factors. This control is obtained by multiple mechanisms, such as different methylation of cytidine-guanosine (CpG) islands in the promoter sites, acetylation-deacetylation of lysine residues of core histones in the nucleosome and production of miRNAs which bind to complementary sequences in the 3’ end of mRNA and interfere with protein synthesis (**Figure 1**) (Goldberg et al., 2007).

### Animal Evidence

Several animal models of fetal growth retardation have been developed to investigate the mechanisms of intrauterine programming, including maternal stress, hypoxia, glucocorticoid treatment, nutrient (protein) restriction, and utero-placental insufficiency.

The most commonly used animal model is based on fetal undernourishment, which may be induced by a complete maternal insufficiency of nutrients or by protein restriction in an isocaloric diet. Intrauterine malnutrition has been proven to change the expression of multiple genes involved in different metabolic pathways (Morris et al., 2009). These changes affect lipid and glucose metabolism, leading the organism to the preferential use of fatty acids as energy source in order to adapt the organism to a reduced nutrient supply.

#### Uteroplacental Insufficiency and Liver Programming

All organs may be affected by intrauterine programming thereafter permanently changing their structure and function. Liver is a major target of programming. Uteroplacental insufficiency alters the expression of genes encoding enzymes involved in hepatic energy production (Lane et al., 1996), decreasing hepatic oxidative phosphorylation (Ogata et al., 1990) and affecting liver glucose transport (Lane et al., 1999).

We investigated the effects induced by an adverse intrauterine environment, using an animal model (Sprague-Dawley rats) of intrauterine growth restriction obtained through maternal uterine artery ligation on day 19 of gestation (Puglianiello et al., 2007; Germani et al., 2008; Puglianiello et al., 2009; Deodati et al., 2018). The animals exposed to uteroplacental insufficiency show an adaptive response in hypothalamic lipid sensing signaling, ultimately influencing food intake and endogenous carbohydrate production in post-natal life (Puglianiello et al., 2009; Deodati et al., 2018). Vuguin et al. (2004) have reported in the same animal model a change in the expression of the genes involved in gluconeogenesis (PEPCK and glucose 6-phosphatase), permanently influencing glucose metabolism in the offspring. These alterations were observed before the development of obesity and diabetes, suggesting that this dysregulation of hepatic glucose metabolism may represent an early defect that contributes to the subsequent onset of fasting hyperglycemia and ultimately diabetes.

The analysis of the liver expression profile of 84 gene showed that 26 genes were differentially expressed in IUGR *versus* SHAM rats. The functional analysis of these genes showed that most of them have a key role in metabolic signaling. In particular, glucose metabolism resulted to be affected by intrauterine malnutrition as indicated by the downregulation of Fbp1, Gpd, Pklr, and the upregulation of Gck, Hk2, and Slc2a1 genes (Cianfarani et al., 2012). Furthermore, in male IUGR adult animals, defective insulin signaling, liver focal steatosis, periportal fibrosis, and chronic activation of hepatic unfolded protein response (UPR) have been described (Deodati et al., 2018).

#### PGC-1 and CPTI

MacLennan et al., 2004 reported higher levels at birth of S-adenosylhomocysteine in the liver of animals exposed to adverse intrauterine environment. These levels were associated with reduced methylation and increased acetylation of histone H3 on lysine 9 (H3K9), lysine 14 (H3K14), and lysine 8 (H3K18) (MacLennan et al., 2004). These modifications persisted up to 21 post-natal day, suggesting a permanent effect on hepatic gene expression.

IUGR rats showed an increased acetylation on H3 in the liver associated with a reduction of nuclear protein levels of histone deacetylase 1 (HDAC1) and HDAC activity. These alterations influence the histone association with the promoter site of PPAR-gamma coactivator (PGC-1) and carnitine-palmitoyl-transferase I (CPTI), two genes whose expression is changed in IUGR rats (Liguori et al., 2010; Puglianiello et al., 2007), PGC-1 expression being upregulated, whereas CPTI expression being downregulated in IUGR rats predisposed to diabetes (Lane et al., 2001; Lane et al., 2002).

PGC-1 is a potent transcription factor that plays a key role in the regulation of cellular mitochondrial function, gluconeogenesis and glucose transport, glycogenolysis, fatty acid oxidation, peroxisomal remodeling, muscle fiber-type switching, and oxidative phosphorylation (Yoon et al., 2001; Lin et al., 2005; Corona et al., 2015). CPTI is a part of the carnitine shuttle and is considered to be a rate-limiting transporter in mitochondrial fatty acid β-oxidation (McGarry and Brown, 1997; Fu et al., 2004). Uteroplacental insufficiency affects the association between acetylated H3/K9 and the promoters of PGC-1 and CPTI, respectively, in IUGR liver, this effect persisting up to day 21 of life in male animals (Fu et al., 2004).

#### Intrauterine Programming and Pancreatic β-Cell

Adverse intrauterine environment may induce permanent and progressive alterations in the gene expression of susceptible organs, including pancreatic islets. DNA methylation of beta cell genome in animal models of uteroplacental insufficiency was investigated (Thompson et al., 2010). A genome-wide HpaII tiny fragment enrichment by ligation-mediated PCR assay (Oda M., et al., 2009 *Nucleic Acids Res*), generating DNA methylation map at almost 1 million loci in IUGR and SHAM animals, was used. In male IUGR rats a different cytosine methylation pattern in approximately 1,400 loci was found. The epigenetic changes mainly occurred in conserved intergenic sequences, located near genes regulating key processes, such as vascularization, β-cell proliferation, insulin secretion, and cell death, and were associated with concordant changes in mRNA expression. These findings suggest that epigenetic dysregulation may be a mechanism involved in propagating the biological memory of intrauterine events, leading to altered expression of nearby genes and ultimately affecting the susceptibility to type 2 diabetes (Thompson et al., 2010) (**Table 1**).

**Table 1:** The relationship between genes involved *in utero* programming and development of metabolic disease.

*Ref*	*Model*	*Organ*	*Gene*	*Epigenetic change*	*Gene function*
Fu et al., 2004	Rats Uteroplacental insufficiency	Liver	PPAR-yCo CPT-I	H3K9Hypetacetylation affecting with gene promoter	Transcriptional coactivator of key gluconeogenic enzymesRate-limiting transporter in mithocondrial fatty acid *β*-oxidation

Park et al., 2008	Rats Uteroplacental insufficiency	Pancreatic islets	PDX-1	H3 and H4Deacetylation, H3H4demetlylation, H3K9 methylation	Transcription factor critical for *β*

Raychaudhuri et al., 2008	Rats Caloric restriction	Skeletal muscle	GLUT4	H3K14 deacetylation,H3K9 methylation	Glucose transporter

Thompson et al., 2010	Rats Uteroplacental insufficiency	Pancreatic islets	CGH-1FGFR-1PCSK -5	CpG hypermethylation in intergenic sequences (IS) CpG hyp omethylation in IS CpG hypmnethylation in transcription start site	Role in endothelial dys function and *β*-cell developmentFibrobalst growth factor receptorRole in peptide processing and maturation

Suter et al., 2012	Rats HFD	Liver	SIRT-1	H3K14 Hyperacetylation	Regulation of glucose homesotasis insulin sensitivity, oxidative stress and anti- inflammatory activity

Heijmans et al., 2008	Humans AGA, periconceptionale famine	Blood	IGF -2	CpG hypomethylation	Fetal growth

Barres et al., 2009	Humans AGA Incubation with TNF alpha,FFA, glucose	Skeletal muscle	PPAR-y-C1 a1pha	Non CpG hypermethylation	Transcriptional Coactivator.regulator of mithocondrial genes

Brøns et al., 2010	Humans SGA/AGA HFD	Skeletal muscle	PPAR -y-C1 a1pha	CpG hypermethylation	Transcriptional Coactivator,regulator of mithocondrial genes

AGA, adequate gestational age; CGH-1, GTP cyclohdrolase1; CPT-1, carnitine-palmitoyl transferase I; FGFR-1, fibroblast growth factor receptor 1; FFA, free fatty acid; GLUT-4, glucose transporter 4; HFD, high fat diet; IGF-2, insulin-like growth factor 2; PCSK-5, proprotein convertase subtilisin/ketin type 5; PDX-1, pancreatic and duodenal homeobox1; PPAR-y coactivator, peroxisome proliferator-activated receptor-gamma coactivator; SGA, small for gestational age; SIRT-1, sirtuin 1 deacetylase; TNF alpha, tumor necrosis factor alpha.

#### Pdx1

Pdx1 (pancreatic and duodenal homeobox 1) is a transcription factor involved in the regulation of pancreatic development and β-cell differentiation. Experimental evidence has documented that a decreased expression of Pdx1 is associated with β-cell dysregulation and impaired islet compensation in the presence of insulin resistance (Stoffers et al., 1997; Holland et al., 2005). Adverse intrauterine environment may lead to different epigenetic modifications of Pdx1 gene expression, such as histone modifications, DNA methylation, and chromatin remodeling (Park et al., 2008). In particular, decreased mRNA level of Pdx1 associated with normal β-cell mass was observed at birth in IUGR pups. In adult life, IUGR animals showed a progressive reduction of β-cell mass associated with undetectable mRNA level of Pdx1. These changes were associated with a significant decrease in H3 and H4 acetylation, a decreased methylation on H3K4, and a significant increase of methylation on H3K9 in Pdx1 proximal promoter of IUGR islets. Overall, these changes resulted in a chromatin silencing, with reduced USF-1 (a key transcription factor) binding and increased engagement of HDAC1 and its corepressor Sin3A.

Postnatally, these epigenetic changes and the reduction in Pdx1 expression can be reversed by HDAC1 inhibition. However, as H3K9me2 accumulates, DNMT3A (a DNA methyltransferase) is recruited to the promoter and initiates *de novo* DNA methylation, which silences pancreas function, eventually leading to diabetes (Park et al., 2008; Pinney and Simmons, 2010; Liguori et al., 2010) (**Table 1**).

#### Intrauterine Programming and Skeletal Muscle

##### GLUT4

*In utero* malnutrition induces histone modifications in skeletal muscle that directly influence the expression of GLUT type 4 (GLUT 4) (Raychaudhuri et al., 2008). GLUT4 is one isoform of a family of sugar transporter proteins containing 12-transmembrane domains with the function of glucose carriers and plays a key role in glucose homeostasis, depending on insulin action. In skeletal muscle of female rats at 450 days of post-natal life a significant reduction of GLUT4 expression was found. This gender-specific difference may originate from early alterations in pancreatic β-islet cell function, occurring during fetal development (Chamson-Reig et al., 2006). Young females showed lower post-natal insulin levels. This IUGR model showed deacetylation and di-methylation of amino acid residues in the N-tail of histone 3. These results suggest that epigenetic histone modification may influence GLUT4 transcription in skeletal muscle of adult female IUGR rats.

#### Maternal Protein Restriction and Offspring Programming

Intrauterine programming may be caused by maternal protein restriction during pregnancy. In pregnant rats, protein deficiency leads to structural and functional changes in liver of offspring, altering glucose homeostasis and insulin sensitivity (Burns et al., 1997; Desai et al., 1997). In young adult male rats, maternal nutritional restriction during pregnancy influences the expression of 249 genes, affecting the development of adiposity and insulin resistance (Morris et al., 2009). In particular, these animals show downregulation of genes involved in intracellular uptake of glucose and its metabolism *via* the glycolytic and tricarboxylic acid signaling, and upregulation of genes involved in the intracellular trafficking and oxidation of fatty acids (Morris et al., 2009). Multiorgan transcriptional modifications in the offspring are induced by maternal protein restriction causing a rearrangement of transcription factor-binding sites, especially in the liver (Vaiman et al., 2011).

#### Maternal Hypoxia and Programming

IUGR may also be the consequence of exposure to reduced oxygen supply *in utero* (Camm et al., 2011). Intrauterine hypoxia induces fetal adaptations in developing organs (kidneys, heart, and vascular system), leading to reduced nephron number and glomerular filtration and ultimately to hypertension in the offspring (Silver et al., 2003). Dysregulation also involves the function of the renin angiotensin system (RAS) *via* altered expression and activation of angiotensin receptor subtypes which influence renal and vascular function (Vehaskari et al., 2001).

IUGR offspring, caused by fetal hypoxia, show altered vasoconstrictor and vasodilator mechanisms together with vascular remodeling (Morton et al., 2016).

These effects of *in utero* hypoxia on offspring cardiometabolic risk may be mediated by epigenetic changes. Increased DNA methylation subsequent to fetal hypoxia causes the reduction of protein kinase Cε (PKCε) gene expression in the heart of pups, resulting in higher cardiac susceptibility to ischemic injury (Patterson and Zhang, 2010).

In placentas of women with pregnancies complicated by IUGR, a vascular dysfunction was found, suggesting a key role played by placental oxidative stress (Schneider et al., 2015).

Fetal programming in hypoxic or complicated pregnancies may be avoided with the use of antioxidant treatments. For instance, the early use of resveratrol [4 g/kg diet, gestational day (GD) 0.5–21] reduced blood pressure in hypertensive rats (Care et al., 2016). The use of ascorbic acid (5 mg/ml in drinking water, GD 6–20) was shown to inhibit placental oxidative stress related to maternal hypoxia (Richter, et al., 2012). However, results from prenatal antioxidant treatments are conflicting (Giussani et al., 2012; Stanley et al., 2012).

#### Maternal Overnutrition and Programming

Intrauterine overnutrition could be associated with the development of metabolic diseases in the offspring (Lillycrop, 2011; Pasternak et al., 2013). A maternal high fat diet (HFD) may cause changes in hypothalamic gene expression in the offspring, such as leptin receptor, proopiomelanocortin, and neuropeptide Y (Chen et al., 2008). Maternal HFD has been reported to increase the acetylation of histone H3 in the offspring (Aagaard-Tillery et al., 2008) and modify methylation and expression of the genes of the mesocortico-limbic reward circuitry (dopamine and opioids).

SIRT1, a member of the HDAC family, influences fat metabolism in adipocytes by repressing PPAR-gamma; upregulation of SIRT1 increases lipolysis, thereby inducing fat loss. SIRT1 is also implicated in the control of glucose homeostasis, insulin sensitivity, oxidative stress, and anti-inflammatory activity. Maternal HFD has been shown to reduce SIRT1 expression in fetal liver and heart by increasing the acetylation of histone H3K14 (Suter et al., 2012).

### Human Evidence

Epidemiological studies have linked a suboptimal intrauterine environment and low birth weight with cardiometabolic risk in adult life (Hales et al, 1991; Ravelli et al, 1998). Notably, metabolic risk is mainly related to the pattern of early growth. The relationship between early catch-up growth and several determinants of cardiovascular disease and type 2 diabetes in adulthood has been reported (Cianfarani et al., 1999; Leunissen et al., 2009; Mericq, et al., 2017). Infants born small for gestational age (SGA) show a decrease in absolute fat mass when compared with appropriate for gestational age (AGA) children, reflecting reduced intrauterine fat accumulation (Ibanez et al., 2010). On the contrary, children exposed to uteroplacental insufficiency show an increase in fat accumulation when these babies experience rapid catch up growth during early postnatal life. SGA infants who achieve normal weight and height within 2 years of age show an increase of total body and abdominal fat between 2 and 4 years of age compared with AGA children (Ibanez et al., 2006).

#### IGF2

The possible involvement of epigenetic mechanisms in the development of human diseases, such as type 2 diabetes, was first suggested by data collected from subjects who were prenatally exposed to famine during the Dutch Hunger Winter in 1944–45. During this period, due to the food embargo imposed by Germany, famine was experienced in the western part of the Netherlands with an average daily ration of 667 kcal. Analysis of data from these individuals revealed an increased risk of developing cardiometabolic disease in adulthood (Lumey et al., 2007).

Notably, a reduced DNA methylation of the imprinted IGF2 gene was reported in a cohort of these subjects exposed to the famine during the prenatal period and evaluated six decades later (Heijmans et al., 2008). Therefore, permanent epigenetic modifications may result from the early exposure to malnutrition during critical time windows of development (Heijmans et al., 2008; Tobi et al., 2012). Additionally, a higher risk of insulin resistance was found in the daughters of women exposed to the Dutch Hunger Winter, suggesting that programming might be transmitted to the following generations (Stein and Lumey, 2000).

The maternal intake of protein and folic acid before and during pregnancy has been related to the degree of IGF2 methylation. A recent study, conducted in 120 children (aged 17 months), evaluated the degree of IGF2 methylation according to exposure (n = 86) or not (n = 34) to folic acid supplementation to mothers in the periconceptional period. The children exposed to folic acid showed higher methylation degree of the IGF2 *differentially methylated region* (DMR) compared with those not exposed. Interestingly, IGF2 DMR methylation was associated with mother’s S-adenosylmethionine blood levels. Additionally, IGF2 DMR methylation was inversely related to birth weight (Steegers-Theunissen et al., 2009).

In summary, the use of folic acid during the peri-conception period is correlated with epigenetic modifications of IGF2 in children, potentially inducing consequences in adult life related to intrauterine programming (Steegers-Theunissen et al., 2009; Haggarty, 2013).

#### PPAR-γC1-α

PGC-1 family of co-activators is influenced by multiple environmental and nutritional factors.

These coactivators play a key role in the regulation of glucose and lipid metabolism and body energy expenditure. Alterations in PGC-1 mediated mechanisms affect multiple metabolic pathways, leading to development of chronic disease (Finck and Kelly, 2006). Muscle and pancreatic islets of individuals with type 2 diabetes show an epigenetic regulation of the transcriptional coactivator peroxisome proliferator activated receptor γ coactivator-1 α (protein PGC-1α; gene PPAR-γC1-α), characterized by increased DNA methylation in PPAR-γ-C1-α gene promoter (Ling et al., 2008). Barres et al. (2009) performed a genome-wide promoter analysis of DNA-methylation to assess genes differentially methylated in skeletal muscle of normal glucose-tolerant and T2DM subjects. In this study, they found cytosine hypermetylation of genes involved in mitochondrial functions, such as PPAR- γ-C1-α in diabetic subjects. Methylation levels were negatively associated with PGC-1α and mitochondrial DNA. Furthermore, the authors provided a mechanism by which high level of cytokines, glycemia, or lipids can induce non-CpG methylation of the PGC-1α promoter in skeletal muscle. To investigate whether these external factors could directly influence the methylation status, they incubated primary human skeletal muscle cultures with multiple factors known to induce insulin resistance, such as hyperglycemia, hyperinsulinemia, elevated free fatty acids (FFA), and elevated cytokines. In these conditions an increase of non-CpG methylation in human myotubes, exposed to fatty acids and TNF-alpha, were observed. Moreover, selective silencing of DNA methyltransferase-3B prevented palmitate-induced PGC-1α promoter methylation.

These findings suggest that environmental changes can alter epigenetic modulation of PGC-1α, which is involved in T2DM and metabolic disease.

Additionally, PPARγ-C1-α promoter methylation status is stable in the blood of 5–7 year old children, representing a marker of fat accumulation up to 14 years of age (Clarke-Harris et al., 2014), thus suggesting that epigenetic analysis may be helpful in identifying individuals at risk of later onset of obesity and cardiometabolic disease (**Table 1**).

### miRNA

#### Animal and Human Evidence

miRNAs have been related to the development of metabolic disease. MicroRNAs are small, non-coding, highly conserved regulatory RNAs (Bartel, 2004). miRNAs can regulate gene expression at a post-transcriptional level, and are implicated in multiple human process, including proliferation, differentiation, development, metabolism, and apoptosis (Lynn, 2009).

Recently, miRNAs have emerged as important regulators of several physiological and pathological processes. For instance, miR-199a may play a role in the development of liver and neurologic disease, by regulating hypoxia-inducible factor-1 alpha (Jiang et al., 2014).

Different miRNAs are associated with several metabolic pathways, including lipid metabolism, glucose metabolism, food intake, body weight homeostasis, inflammation, oxidative stress, expression of several cytokines, and angiogenesis in obesity. Intrauterine programming can cause modifications in miRNA expression, inducing alterations of metabolic pathways in children born to mothers affected by obesity (Williams, 2012). Circulating microRNAs (c-miRNAs) may reflect these alterations in tissue expression and intracellular signaling, supporting their utility as informative biomarkers.

miR-141 resulted significantly up-regulated in both the placenta and plasma of women affected by uteroplacental insufficiency and may play a key role in the pathogenic mechanism of IUGR by suppressing pleomorphic adenoma gene 1 (PLAG1) (Tang et al., 2013). PLAG1 is expressed mainly in placenta during embryonic development. In animal model, the disruption of PLAG1 causes growth retardation (Braem et al., 2002). Microarray analyses identified genes that were induced or repressed by PLAG1. In particular, this analysis showed an upregulation of genes encoding growth factors, such as insulin like growth factor 2 (IGF2), bone-derived growth factor (BPGF1), vascular endothelial growth factor (VEGF), and placental growth factor (PGF) (Van Dyck et al., 2004).

These findings are consistent with data reported by Tang et al. (2013) who found a significant positive correlation between mRNA expression of PLAG1 and level of IGF2 in placental tissues of fetal growth retardation compared to controls. Moreover, a downregulation of miR-16 and miR-21 in the placenta was significantly related with lower birth weight (Maccani and Marsit, 2011).

miRNA serum profile in umbilical cord of SGA children with (SGA-CU, n = 18) and without catch-up growth (SGA-nonCU, n = 24) was investigated (Mas-Parés et al., 2018). In particular, 12 miRNA were differentially expressed between SGA-CU and SGA-nonCU (miR-128-3p, miR-222-5p, miR-300, miR-374b-3p, miR-501-3p, miR-548c-5p, miR-628-5p, miR-770-5p, miR-873-5p, miR-876-3p and miR-940). Among them, miR-501-3p, miR-576-5p, miR-770-5p, and miR-876-3p showed a significant association with weight, height, weight catch-up, and height catch-up at 1 year of age in all subjects. mir-576-5p was an independent predictor of weight, waist circumference, and renal fat at 6 years of life. Additionally, *in silico* analysis revealed that mir-576-5p was associated with key metabolic pathways, such as insulin, IGF1, PDGFR-B, and mammalian target of rapamycin (mTOR) signaling. These findings suggest that miR-576-5p could play a role for the risk of metabolic disease related to postnatal growth.

MicroRNA expression in newborns with different birth weight (normal birth weight *vs.* low birth weight *vs.* high birth weight) was also reported. miR-454-3p was upregulated in low and high birth weight newborns compared to normal birth weight newborns. The analysis of prediction target genes revealed that seven possible pathways could be regulated by this microRNA, such as endocytosis, transforming growth factor ß (TGF-ß), axon guidance, forkhead box O (FoxO), p53, proteoglycans in cancer, and Hippo signaling pathway (Rodil-Garcia et al., 2017).

miRNAs are associated to metabolic disease such as insulin resistance, obesity, and non-alcoholic fatty liver disease (NAFLD). In particular, an increasing body of evidence shows that almost 100 different miRNAs are differentially expressed in non-alcoholic steato-hepatitis (NASH) patients (Cheung et al., 2008).

A high fructose diet administered to induce IR in mice led to overexpression of several miRNAs such as miR-19b-3p, miR-101a-3p, miR-30a-5p, miR-582-3p, and miR-378a-3p, and downregulation of miR-223-3p, miR-33-5p, miR-128-3p, miR-125b-5p, and miR-145a-3p.

Interestingly, IRS-1, FOXO-1, SREBP-1c, SREBP-2, SREBP-2, Insig-1, and Insig-2 (Ing-2a and -2b), which are involved in insulin pathway and synthesis of hepatic lipids, are target genes of these miRNAs (Sud et al., 2017).

Another study found 46 distinctively expressed miRNAs in patients affected by NASH. Twenty-three resulted overexpressed, such as miRNA-21, miRNA-100, and miRNA-34a, and others including miRNA-126 and miRNA-122 were down-regulated (Cheung et al., 2008). Offspring of mothers exposed to HFD during gestation and lactation showed an altered expression of 23 miRNAs in the liver (Zhang et al., 2009). Specifically, miRNA122, the main hepatic miRNA, resulted downregulated in subjects affected by NAFLD (Zhang et al., 2009) and its deletion in mice caused hepatic steatosis, inflammation, and hepatocellular carcinoma. Therefore, miRNA-122 has been suggested as a major contributor to the regulation of hepatic lipid metabolism (Wen and Friedman, 2012). Additionally, mir-146b, mir-143, mir-34a, and mir-23a resulted increased in NAFLD patients (Tian et al., 2013). MiRNA 370, 33, 103, and 104 have also been recognized to modulate lipid and cholesterol regulatory genes, contributing to the development of NAFLD. Mice with deletion of this liver-specific miRNAs developed hepatic steatosis, inflammation, and hepatocellular carcinoma (Wen and Friedman, 2012).

miRNAs 146b, 143, 34a, and 23a have been found upregulated in NAFLD (Tian et al., 2013). Other miRNAs have been reported to influence lipid and cholesterol regulatory genes (miRNA 370, 33, 103, and 104), eventually contributing to the development of NAFLD.

We investigated hepatic miR-122-5p RNA expression in an animal model of IUGR induced by uterine artery ligation. Twelve SHAM and 12 IUGR male rats from four different litters were randomly selected. IUGR pups showed significant upregulation of gluconeogenesis and lipogenesis genes. Significantly reduced glucose tolerance and liver focal steatosis with associated periportal fibrosis were observed in adult IUGR male rats (Deodati et al., 2018). However, no significant difference in hepatic miR-122-5p RNA expression was observed.

A recent study, comparing obese SGA and AGA children with normal weight SGA and AGA children has shown a specific profile of circulating miRNAs (Marzano et al., 2018). Twenty eight miRNAs resulted dysregulated in obese-SGA (OB-SGA) *vs.* normal weight-SGA (NW-SGA) and 19 miRNAs were altered in OB-AGA *vs.* NW-AGA. In particular, miR-92a-3p, miR-122-5p, miR-423-5p, miR-484, miR-486-3p, and miR-532-5p were up-regulated, and miR-181b-5p was down-regulated in both OB-SGA and OB-AGA compared with normal weight subjects. These miRNAs resulted implicated in insulin signaling, glucose trafficking, insulin resistance, and lipid metabolism (**Table 2**). More specifically, an association between miR-122-5p and obesity, lipid metabolism, insulin resistance, metabolic syndrome, and type 2 diabetes (Wang et al., 2015) was found whereas mir-486-3p is increased in pre-diabetes and obesity. A significant association was found between circulating levels of miR-486-5p, miR-486-3p and miR-423-5p and BMI, fat mass, waist circumference, regional fat distribution, homeostatic model assessment of insulin resistance (HOMA-IR), high-molecular-weight adiponectin, C-reactive protein, and circulating lipids (Prats-Puig et al., 2013). A selective deregulation of miR-26a-5p, miR-126-3p, and miR-143-3p was observed in preeclampsia and IUGR pregnancies (Hromadnikova et al., 2015).

**Table 2:** List of target genes, involved in severe metabolic pathways, differentially influenced by microRNAs expression.

Metabolic Pathway	Target genes
Insulin signaling	AKTl, MAPKl, PDKl, TFRC, GRB2, SOSl, RAFl, EEF2,MTOR, IRSl, PIK3Rl, PTPNll
Glucose transport	AKTl, CDKNlA, PDPKl, TFRC, GRB2, SOSl, EEAl, MTOR, IRSl
Cholesterol and Lipid metabolism	SREBFl, HDLBP, MBTPS2, LDLR, HMGCR, INSIGl, HMGCSl, ERLINl, PRKAAl, ABCAl, LDLRAPl, SREBF2, DHCR24, CYB5R3, SCAP, FDFrl, NPC2, INSIG2
Insulin Resistance	SREBFl, CRTC2, IRS2, PTPRF,PIK3CB, SOCS3, CREBl, TRIB3, FOXOl, PPPlCC, PTEN, PPP1CA,GSK3B, GFPTl, GFPT2, SLC2Al, MGEA5, CREB3L2, PIK3CA, MLXIP, PRKAAl, MAPK8, OGT, NFKBl,RPS6KBl, AKTl, PDPKl, PIK3R3, PIK3Rl, PIK3CG, PRKAB2, PIK3CD, PCK2,PRKCD, IRSl, STAT3, PTPNll, MAPK9, PTPNl, MTOR

AKT1, AKT serine/threonine 1; MAPK1-8-9, mitogen-activated protein kinase 1-8-9; PDK1, pyruvate dehydrogenase kinase 1; TFRC, transferrin receptor; GRB2, growth factor receptor-bound protein 2; SOS1, son of sevenless homolog 1; RAF1, Raf-1 proto-oncogene, serine/threonine kinase; EEF2, eukaryotic elongation factor 2; MTOR, mammalian target of rapamycin; IRS1-2, insulin receptor substrate 1-2; PIK3R1-3, phosphoinositide-3-kinase regulatory subunit 1-3; PTPN11, protein tyrosine phosphatase non-receptor type 11; CDKN1A, cyclin dependent kinase inhibitor 1A; PDPK1,3-phosphoinositide dependent protein kinase 1; EEA1, early endosome antigen 1; SREBF1-2, sterol regulatory element binding transcription factor 1-1; HDLBP, high density lipoprotein-binding protein; MBTPS2, membrane bound transcription factor peptidase, site 2; LDLR, low-density lipoprotein receptor; HMGCR, 3-hydroxy-3-methylglutaryl-CoA reductase; INSIG1-2, insulin induced gene 1-2; HMGCS1,3-hydroxy-3-methylglutaryl-CoA synthase 1; ERLIN1, ER lipid raft associated 1; PRKAR1A, protein kinase cAMP-dependent type I regulatory subunit alpha; ABCA1, ATP-binding cassette transporter; LDLRAP1, low-density lipoprotein receptor adapter protein 1; DHCR24, 24-dehydrocholesterol reductase; CYB5R3, cytochrome B5 reductase 3; SCAP, sterol regulatory element-binding protein cleavage-activating protein; FDFT1, farnesyl-diphosphate farnesyltransferase 1; NPC2, NPC intracellular cholesterol transporter 2; CRTC2, CREB regulated transcription coactivator 2; PTPRF, protein tyrosine phosphatase receptor type F; PIK3CB-A-G-D, phosphatidylinositol-4,5-bisphosphate 3-kinase catalytic subunit beta-alpha-gamma-delta; SOCS3, suppressor of cytokine signaling 3; CREB1, CAMP responsive element binding protein 1; TRIB3, tribbles homolog 3; FOXO1, forkhead box O1; PPP1CC-A, protein phosphatase 1 catalytic subunit gamma; PTEN, phosphatidylinositol 3,4,5-trisphosphate 3-phosphatase and dual-specificity protein phosphatase; GSK3B, glycogen synthase kinase 3 beta; GFPT1-2, glutamine–fructose-6-phosphate transaminase 1-2; SLC2A1, solute carrier family 2 member 1; MGEA5, meningioma expressed antigen 5; CREB3L2, CAMP responsive element binding protein 3 like 2; MLXIP, MLX interacting protein; PRKAA1, protein kinase AMP-activated catalytic subunit alpha 1; OGT, O-linked N-acetylglucosamine (GlcNAc) transferase; NFKB1, nuclear factor kappa B subunit 1; RPS6KB1, ribosomal protein S6 Kinase B1; PDPK1, 3-phosphoinositide dependent protein kinase 1; PRKAB2, protein kinase AMP-activated non-catalytic subunit beta 2; PCK2, phosphoenolpyruvate carboxykinase 2; PRKCD, protein kinase C delta; STAT3, signal transducer and activator of transcription 3; PTPN1 (protein tyrosine phosphatase, non-receptor type 1).

### Genome Wide Association and Intrauterine Programming

Birth weight is a complex trait influenced by environmental and maternal-fetal genetic factors.

To clarify the association between birth weight and development of metabolic diseases in adult life, genome-wide association studies were performed.

The first meta-analysis of GWA studies reported data on birth weight in 10,623 subjects. The first cluster associated with birth weight includes CCNL1 and LEKR1 genes (Freathy et al., 2010). This cluster of SNPs was correlated also to reduced ponderal index at birth and smaller head circumference and birth length, suggesting that these loci could influence soft tissue rather than skeleton. In literature, there is a strong evidence of relationship between these genes and birth weight but the underlying mechanism has not been elucidated yet. The second cluster of SNPs was centered on the ADCY5 gene. This locus is associated to lower birth weight and predisposition of type 2 diabetes. Strong associations have been reported between the type 2 diabetes risk allele and higher fasting glucose (Dupuis et al., 2010) and higher 2-h glucose level during oral glucose tolerance test (OGTT) with lower 2-h insulin level and HOMA in adults (Saxena et al., 2010). In addition, evidence from studies of other type 2 diabetes loci is accumulating for association between the fetal risk alleles at CDKAL1 and HHEX-IDE and lower birth weight (Andersson et al., 2010). In particular, both the CDKAL1 and HHEX-IDE have been associated with reduced pancreatic beta-cell function and reduced insulin secretion in studies on non-diabetic adults (Pascoe et al., 2007; Steinthorsdottir et al., 2007). Further studies are needed to show whether these loci may influence birth weight by altering intrauterine insulin secretion.

There evidences that all type 2 diabetes loci are not associated with birth weight. For example, TCF7L2, which confers the largest risk of type 2 diabetes in Europe, showed no link *via* the fetal genotype (Mook-Kanamori et al., 2009; Andersson et al., 2010). These findings are in contrast to the effects of ADCY5, CDKAL1, and HHEX-IDE, previously reported, and suggest that there is an overlap between the genetics of type 2 diabetes and intrauterine growth.

Sixty-five loci have been correlated with birth weight in genome-wide association studies (GWASs) (Hattersley and Tooke, 1999; Freathy, 2010; Horikoshi et al., 2016). Most of these studies did not discriminate between maternal and fetal genetic influences, producing overlapping signals due to the correlation between maternal and fetal genotypes.

A recent meta-analysis of GWASs of offspring birth weight, reporting maternal genotypes in up to 86,577 women of European descent from 25 studies identified 10 autosomal loci (MTNR1B, HMGA2, SH2B3, KCNAB1, L3MBTL3, GCK, EBF1, TCF7L2, ACTL9, CYP3A7) that were associated with offspring birth weight (Weedon et al., 2007; Beaumont et al., 2018). In addition, at least 7 of the 10 associations were consistent with effects of the maternal genotype acting *via* the intrauterine environment, rather than *via* shared alleles with the fetus. This study confirms previous results about the associations with birth weight at TCF7L2 and GCK (Freathy, 2010; Freathy et al., 2007).

Another study of GWA evaluated maternal and fetal genetic effects on birth weight and their impact on metabolic risk, using Mendelian randomization to distinguish the contributions of direct fetal and indirect maternal genetic effects (Warrington et al., 2019). They identified 64 SNPs with fetal effects, 32 SNPs with maternal effects, 27 SNPs with directionally concordant fetal and maternal effects, and 15 SNPs with directionally opposing fetal and maternal effects. Furthermore, the authors performed the analysis to estimate the effect of insulin secretion on birth weight, including SNPs previously associated with fasting glucose and insulin sensitivity. They confirmed previous results of GWA SNPs for fasting glucose, T2D, insulin secretion, and insulin sensitivity loci and supported the opposing contributions of fetal *versus* maternal glucose-raising alleles on birth weight.

In conclusion, the Mendelian randomization provided evidence about the influence of fetal insulin on intrauterine growth and the link between lower birth weight with reduced insulin secretion and higher T2D risk in adults (Hattersley and Tooke, 1999; Warrington et al., 2019).

Further experimental and clinical studies are necessary to clarify the link between events *in utero* environment and predisposition to metabolic disease in adult life.

## Conclusion

Although the available data are not conclusive and even partly conflicting, an increasing body of evidence indicates that a suboptimal intrauterine environment induces epigenetic changes and eventually leads to programming. These epigenetic fingerprints are still present at birth and are related to specific structural and functional alterations in postnatal life. The question whether these epigenetic changes are causative or simply associated with the metabolic profile remains unanswered. Nevertheless, the epigenetic modifications, especially those detectable in the circulation, such as miRNAs or SNPs, may become predictive markers of cardiometabolic risks provided that a specific pattern is proven to be associated with long-term risk in controlled prospective longitudinal studies.

## Author Contributions

AD, EI, and SC wrote the article. SC critically reviewed the article.

## Conflict of Interest

The authors declare that the research was conducted in the absence of any commercial or financial relationships that could be construed as a potential conflict of interest.
